# Morally Reframed Arguments Can Affect Support for Political Candidates

**DOI:** 10.1177/1948550617729408

**Published:** 2017-09-28

**Authors:** Jan G. Voelkel, Matthew Feinberg

**Affiliations:** 1 Department of Social Psychology, Tilburg University, Tilburg, the Netherlands; 2 Rotman School of Management, University of Toronto, Toronto, Ontario, Canada

**Keywords:** moral psychology, political psychology, influence, attitudes

## Abstract

Moral reframing involves crafting persuasive arguments that appeal to the targets’ moral values but argue in favor of something they would typically oppose. Applying this technique to one of the most politically polarizing events—political campaigns—we hypothesized that messages criticizing one’s preferred political candidate that also appeal to that person’s moral values can decrease support for the candidate. We tested this claim in the context of the 2016 American presidential election. In Study 1, conservatives reading a message opposing Donald Trump grounded in a more conservative value (loyalty) supported him less than conservatives reading a message grounded in more liberal concerns (fairness). In Study 2, liberals reading a message opposing Hillary Clinton appealing to fairness values were less supportive of Clinton than liberals in a loyalty-argument condition. These results highlight how moral reframing can be used to overcome the rigid stances partisans often hold and help develop political acceptance.

Political elections provide the general populace with a choice between two (or more) candidates who have contrasting viewpoints on how best to serve the people and their needs. These viewpoints, and the candidates who endorse them, often fall into opposing sides of the political spectrum, with one candidate representing a more liberal perspective and the other representing a more conservative perspective. Generally, people’s support for one candidate or the other reflects whether they identify with the liberal or conservative perspective that each candidate embodies (e.g., gallup.com, n.d.).

Despite these ideological allegiances, candidates, their campaigns, and everyday supporters invest substantial time and resources in hopes of persuading those who endorse the other candidate that he or she is the wrong person for the job. Even though this process is commonplace and exorbitant amounts of money are devoted to it (Cummings, 2008), it is largely unknown whether such attempts at persuasion are ever effective and, if they are, what types of arguments have the greatest impact.

In the present research, we explore the possibility that certain types of moral arguments can be an effective strategy for persuading liberals and conservatives to be less attached to the candidate who represents their party and perspective. Specifically, we examine the effectiveness of a technique called “moral reframing” (Feinberg & Willer, 2013, 2015) in the context of the United States 2016 presidential election.

## Morality and Political Attitudes

Morality matters for political attitudes (Janoff-Bulman, Sheikh, & Baldacci, 2008; Morgan, Skitka, & Wisneski, 2010). Liberals and conservatives possess different moral worldviews, and such differences help explain many of the contrasting stances the two sides take (Caprara, Schwartz, Capanna, Vecchione, & Barbaranelli, 2006; Graham, Haidt, & Nosek, 2009; Thorisdottir, Jost, Liviatan, & Shrout, 2007). Recently, researchers mapped the moral domain and found evidence for five moral foundations that form the basis of moral beliefs and judgments (Graham et al., 2011; Haidt & Josephs, 2004). The harm/care foundation is concerned with other’s suffering and the need to prevent and alleviate such suffering. The fairness/cheating foundation relates to justice, equality, and discrimination. The loyalty/betrayal foundation emphasizes the importance of one’s in-group and prioritizing that in-group. The authority/subversion foundation deals with respect for higher ranked individuals as well as adherence to tradition. Finally, the sanctity/degradation foundation is concerned with sacredness and purity and avoiding disgust-evoking behaviors (Haidt, 2007, 2012; Haidt & Joseph, 2004). Research has, in turn, found that compared to conservatives, liberals more strongly endorse the harm/care and the fairness/cheating foundations, while conservatives more strongly endorse the loyalty/betrayal, authority/subversion, and sanctity/degradation foundations (Graham et al., 2009; Haidt & Graham, 2007).

## Moral Reframing and Candidate Arguments

Building on this understanding of the moral divide between liberals and conservatives, recent research has shown that it is possible to capitalize on these distinctions for purposes of political persuasion and coalition formation by using “moral reframing” (Day, Fiske, Downing, & Trail, 2014; Feinberg & Willer, 2013, 2015; Kidwell, Farmer, & Hardesty, 2013; Wolsko, Ariceaga, & Seiden, 2016). Moral reframing involves framing arguments that favor one’s own political stance but grounding these arguments in moral terms that appeal to the moral values of those on the other side of the political spectrum. In this research, while liberals were unmoved by arguments in favor of conservative policies grounded in the more conservative moral foundations, their support for the conservative positions increased after reading messages grounded in the more liberal foundations, and this research also demonstrated the reverse when it comes to liberals persuading conservative targets (Feinberg & Willer, 2013, 2015; Kidwell et al., 2013; Wolsko et al., 2016; cf. Day et al., 2014).

Although this past research has shown that moral reframing can be an effective strategy for persuading those on the other side of the political spectrum to be more supportive of policies they would typically oppose, no research has explored the effectiveness of moral reframing in one of the most contentious, but fundamental, political domains—political campaigns. Might moral reframing be an effective means for affecting support for political candidates? We expected that it would, because moral evaluations are particularly relevant for person perception and impression formation overall (Goodwin, Piazza, & Rozin, 2014), and are especially relevant when making judgments about powerful figures and political candidates (Chen, Jing, & Lee, 2012; Skitka & Bauman, 2008; Trevino, Hartman, & Brown, 2000).

Additionally, moral reframing research has primarily focused on the effectiveness of morally reframed messages in support of a stance and has largely not explored whether this technique would work when arguments are made in opposition to a stance. Even so, understanding moral reframing’s effectiveness in decreasing a target’s support is particularly important, considering how much political rhetoric aims to decrease support for a policy or a political candidate. We predicted that the same underlying processes will apply regardless of whether a morally reframed message is in favor or in opposition to a stance; as long as, the argument itself is framed in a manner that appeals directly to the moral values of the targets, then those targets should be responsive to it because it fits with their morality.

## The Present Research

We tested our predictions by examining the effectiveness of morally reframed messages in the context of the U.S. presidential election campaign of 2016, presenting participants with short campaign messages in opposition to either Donald Trump (Study 1) or Hillary Clinton (Study 2). In each study, these messages were framed in terms of either a moral value endorsed at higher levels by conservatives (i.e., loyalty) or a moral value endorsed at higher levels by liberals (i.e., fairness). We expected that conservatives would become less supportive of Donald Trump after reading an oppositional message grounded in loyalty values than after reading a message grounded in fairness values. On the other hand, we expected liberals would become less supportive of Hillary Clinton after reading an oppositional message grounded in fairness values than after reading a message grounded in loyalty values.

We did not make any specific predictions regarding how liberals would respond to the anti-Trump messages and how conservatives would respond to the different messages in opposition to Clinton. Although the anti-Trump messages framed in more liberal moral terms might resonate with liberals and the anti-Clinton messages framed in more conservative moral terms might resonate with conservatives, these arguments may still be ineffective because they were aiming to persuade targets to take on a position that, likely, they already held (cf. Day et al., 2014).

## Study 1

In the first study, we presented participants with arguments opposing Donald Trump that were framed in terms of either fairness or loyalty moral concerns. We hypothesized that conservatives in the loyalty argument condition would support Trump less than conservatives in the fairness argument condition, but the moderate and liberal participants would likely be unaffected by our manipulation. We measured support for Donald Trump, our dependent variable, with both attitudes (warmth and acceptance as president) and behavioral intentions (likelihood to vote for Trump) and tested whether the effect of experimental condition on the likelihood to vote for him might be mediated by the attitudes measures.

## Method

### Participants

Based on the past research on moral reframing (e.g., Feinberg & Willer, 2013, 2015), we expected a small effect size (specifically, a *R*^2^Δ of approximately .02 to .03 as a result of including the interaction of political ideology and experimental condition into the regression equation). In order to have enough statistical power, therefore, we estimated a sample size of around 400 participants in each study would be required. In Study 1, 404 participants recruited from the Amazon Mechanical Turk website completed the study. Participants were excluded if they had missing values (*n* = 3) or if they failed an attention check (*n* = 4). Thus, the final sample size consisted of 397 participants (189 male, 207 female, 1 other; *M*_age_ = 37.33, *SD* = 12.94). Participants took part in this study on August 28, 2016, 72 days prior to the 2016 presidential election and were given a small payment for their participation.

### Procedure

Participants learned they would be presented with some information about a candidate for the 2016 presidential election and be asked questions afterward. Participants were then randomly assigned to one of two conditions: the loyalty or fairness argument condition. Both conditions involved presenting participants with a short message arguing against Donald Trump, modeled after actual campaign advertisements. The loyalty message was written so that it would appeal to the loyalty/betrayal moral foundation, incorporating words and phrases representative of that foundation (cf. Graham et al., 2009). For instance, the loyalty message argued that Trump “has repeatedly behaved disloyally towards our country to serve his own interests” and that “during the Vietnam War, he dodged the draft to follow his father into the development business” (for full text, see Supplemental Material). The fairness argument, in contrast, appealed to the fairness/cheating moral foundation and used words and phrases representative of that foundation. For instance, it argued that Trump “openly discriminates against Muslims threatening their rights to be treated with fairness and equality” and that “his unfair statements are a breeding ground for prejudice” (for full text, see Supplemental Material). Each message was accompanied by a picture of Donald Trump further highlighting the corresponding moral value, showing him either next to American soldiers in action (loyalty argument condition) or next to Muslims demonstrating against terrorism (fairness argument condition).

Following the campaign message, participants were asked to summarize the message they just read, which served as an attention check. Two raters coded whether participants’ answers to the attention check indicated that the participants actually read the arguments. The interrater reliability was high (ϕ = .70). We excluded only those participants for which both coders rated the summary as inadequate. Afterward, participants completed 3 measures relating to Donald Trump. *Warmth* was measured with the item: “How warm or cold do you feel toward Donald Trump?” answered on a scale from 0 (*very cold*) to 100 (*very warm*). *Acceptance as President* was measured with the item: “How easy or hard would it be for you to accept Donald Trump as the President of the United States?”, answered on a scale from 0 (*very easy*) to 100 (*very hard*). Finally, *Likelihood to Vote* was measured with the item: “In the upcoming 2016 presidential election, how likely are you to vote for Donald Trump for president?,” answered on a scale from 0 (*very unlikely*) to 100 (*very likely*). The initial position of the slider for all 3 items was at the midpoint of the scales. Finally, participants completed a demographic questionnaire which included a measure of political ideology (“Generally speaking, do you usually think of yourself as conservative, moderate, or liberal?”) with three response categories (conservative, moderate, and liberal).

### Analysis Strategy

We conducted separate multiple regression analyses for the three dependent variables. A dummy variable for moral argument condition (fairness argument as reference group), two dummy variables for political ideology (conservatives as reference group), and the interaction terms of condition and ideology were included as independent variables.

Although we expected different effects of the moral argument condition for the different ideology groups (implying an interaction effect), our main focus was a priori on the simple slopes analyses. To ensure the robustness of our results, we included several robustness checks that consistently supported our results (for details, see Supplemental Material). In addition, we conducted a moderated mediation analysis using Model 8 of Hayes’ Process macro (Hayes, 2013). We included experimental condition as the independent variable, ideology as the moderator, *warmth* and *acceptance as president* as mediators, and *likelihood to vote for Trump* as the dependent variable. A bias-corrected bootstrap estimation approach with 5,000 samples was used to estimate the indirect effects.

## Results

Means and standard deviations of the dependent variables for each condition by ideology group are presented in Table 1.

**Table 1.** Results of Study 1: Means (*SD*s, *n*) for Argument Condition × Participants' Ideology.

Condition	Ideology		
	Conservative	Moderate	Liberal
(a) Warmth			
Fairness argument	61.04 (31.56, 45)	31.87 (30.50, 67)	5.10 (12.63, 83)
Loyalty argument	47.23 (32.65, 40)	30.44 (30.54, 75)	8.00 (14.86, 87)
(b) Acceptance as president			
Fairness argument	65.84 (32.94, 45)	34.55 (34.00, 67)	11.51 (25.53, 83)
Loyalty argument	50.45 (36.74, 40)	35.68 (34.77, 75)	18.60 (31.49, 87)
(c) Likelihood to vote			
Fairness argument	74.62 (31.72, 45)	31.58 (37.56, 67)	2.06 (8.88, 83)
Loyalty argument	55.75 (39.82, 40)	31.13 (38.31, 75)	7.71 (19.64, 87)

*Note*. The acceptance as president measure was recoded so that higher values indicate that participants were more willing to accept Trump as president.

### Warmth

The regression analysis showed a significant interaction effect, Δ*R*^2^ = 0.01, *F*(2, 391) = 3.14, *p* = .044. Simple-slopes analyses indicated that, as expected, conservative participants perceived Trump as less warm in the loyalty argument condition than in the fairness argument condition, *b* = −13.82, *t*(391) = −2.53, *p* = .012, 95% confidence interval (CI) = [−24.58, −3.06]. There was no significant effect of the moral argument condition for either moderates, *b* = −1.43, *t*(391) = −0.34, *p* = .736, 95% CI [−9.75, 6.90], or liberals, *b* = 2.90, *t*(391) = 0.75, *p* = .453, 95% CI [−4.69, 10.50].

### Acceptance as President

The regression analyses showed a significant interaction effect, Δ*R*^2^ = .01, *F*(2, 391) = 3.48, *p* = .032. Simple-slopes analyses indicated that, as expected, conservative participants accepted Trump less as president in the loyalty argument condition than in the fairness argument condition, *b* = −15.39, *t*(391) = −2.20, *p* = .028, 95% CI [−29.14, −1.65]. There was no significant effect of the moral argument condition for either moderates, *b* = 1.13, *t*(391) = 0.21, *p* = .835, 95% CI [−9.51, 11.76], or liberals, *b* = 7.09, *t*(391) = 1.44, *p* = .152, 95% CI [−2.62, 16.80].

### Likelihood to Vote for Trump

**Figure 1.**
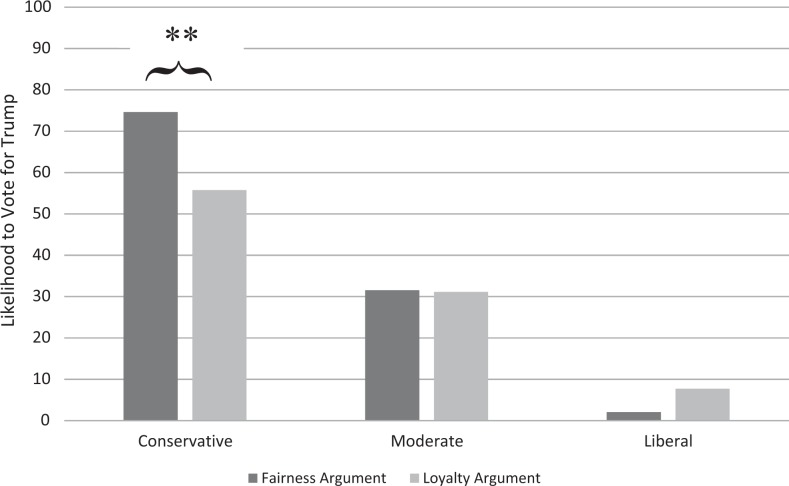
Likelihood to vote for Trump depending on argument condition and participants' ideology. *p < .05, **p < .01.

The interaction effect was significant, Δ*R*^2^ = .02, *F*(2, 391) = 4.84, *p* = .008. Simple-slopes analyses indicated that, as expected, conservative participants were less likely to vote for Trump in the loyalty argument condition than in the fairness argument condition, *b* = −18.87, *t*(391) = −2.91, *p* = .004, 95% CI [−31.61, −6.14]. There was no significant effect of the moral argument condition for either moderates, *b* = −0.45, *t*(391) = −0.09, *p* = .929, 95% CI [−10.30, 9.40], or liberals, *b* = 5.65, *t*(391) = 1.24, *p* = .217, 95% CI [−3.34, 14.65]. These findings are illustrated in Figure 1.

### Moderated Mediation Analyses

The results of the moderated mediation analysis were consistent with our hypotheses. For conservatives, the effect of experimental condition on the likelihood to vote for Trump was mediated by warmth, *b* = −12.15, *SE* = 6.26, 95% CI [−25.07, −0.56], and by acceptance as president, *b* = −1.73, *SE* = 1.07, 95% CI [−4.69, −0.22], and the direct effect of experimental condition on likelihood to vote for Trump was not significant for conservatives, *b* = −4.99, *t*(389) = −1.36, *p* = .173, 95% CI [−12.19, 2.21]. For moderates, there was no indirect effect of experimental condition on the likelihood to vote for Trump, for warmth: *b* = −1.25, *SE* = 4.54, 95% CI [−10.19, 7.75], or for acceptance as president: *b* = 0.13, *SE* = 0.70, 95% CI [−1.27, 1.61]. The direct effect of experimental condition on likelihood to vote for Trump was also not significant for moderates, *b* = 0.68, *t*(389) = 0.24, *p* = .810, 95% CI [−4.85, 6.20]. Likewise, for liberals, there was no indirect effect of experimental condition on the likelihood to vote for Trump, for warmth: *b* = 2.55, *SE* = 1.88, 95% CI [−1.15, 6.32], or for acceptance as president: *b* = 0.80, *SE* = 0.56, 95% CI [−0.04, 2.27], and the direct effect of experimental condition on likelihood to vote for Trump was not significant for liberals, *b* = 2.30, *t*(389) = 0.90, *p* = .371, 95% CI [−2.75, 7.36].

## Discussion

We found causal evidence that, compared to arguments in opposition to Donald Trump grounded in fairness concerns, arguments opposing Trump that appealed to the more conservative value of loyalty were more effective in causing conservative participants to feel colder toward Trump, to accept him less as president, and, most importantly, to be less likely to vote for him. Further, the results suggest that the effect of moral argument condition on the likelihood to vote for Trump was mediated by perceived warmth and acceptance as president for conservatives. We did not find convincing evidence that the moral argument condition affected the support of moderates or liberals for Donald Trump.

## Study 2

In Study 2, we aimed to conceptually replicate Study 1 with Hillary Clinton as the target instead of Donald Trump. That is, we presented participants with arguments opposing Hillary Clinton’s candidacy that were framed in terms of either fairness or loyalty moral concerns. We hypothesized that liberals in the fairness argument condition would support Clinton less than liberals in the loyalty argument condition, while the manipulation would not affect the moderates or conservatives. We measured support for Hillary Clinton with the same measures as in Study 1 and tested whether the effect of experimental condition on the likelihood to vote for her would be mediated by the attitudes measures.

## Method

### Participants

Four hundred and eight participants recruited from the Amazon Mechanical Turk website completed the study. Participants were excluded if they had missing values (*n* = 3) or if they failed the attention check (*n* = 13). Thus, the final sample size consisted of 392 participants (172 males, 218 females, 1 agender, 1 genderqueer; *M*_age_ = 36.86, *SD* = 12.24). Participants took part in this study on September 2, 2016, 67 days prior to the 2016 presidential election and were given a small payment for their participation.

### Procedure

The procedure paralleled that of Study 1, except the target of the message this time was Hillary Clinton instead of Donald Trump. Accordingly, we formulated messages in opposition to Clinton grounded in either loyalty or fairness values. For instance, the loyalty message argued that Clinton “is willing to risk the standing of our nation to achieve her own goals” and that “she failed our ambassador and soldiers in Benghazi” (for full text, see Supplemental Material). The fairness argument, in contrast, argued that “while so many Americans have suffered during the recent recession that the Wall Street Banks helped cause, Clinton has accepted millions of dollars from them in exchange for giving a few speeches” and that “Clinton is willing to sacrifice fairness and equality to achieve her own goals” (for full text, see Supplemental Material). The loyalty argument was accompanied by a picture showing Hillary Clinton next to an open envelope with an email symbol inside. The fairness argument was accompanied by a picture showing Hillary Clinton next to a Wall Street sign.

Following the campaign message, participants were asked to summarize the message they just read. As in Study 1, two raters coded whether participants’ answers to the attention check indicated that the participants actually read the arguments. The interrater reliability was high (ϕ = .89). We excluded only those participants for which both coders rated the summary as inadequate. Afterward, they completed the same three measures that were used in Study 1 regarding Hillary Clinton (warmth, acceptance as president, likelihood to vote). At the end of the study, participants completed a demographic questionnaire which included the same measure of political ideology as used in Study 1.

### Analysis Strategy

We used the same analysis strategy as in Study 1 except that this time the loyalty condition was used as reference category for the moral argument manipulation. Again, we used several robustness checks that consistently supported our results (for details, see Supplemental Material).

## Results

Means and *SD*s of the dependent variables for each condition by ideology group are presented in Table 2.

**Table 2.** Results of Study 2: Means (*SD*s, *n*) for Argument Condition × Participants' Ideology.

Condition	Ideology		
	Conservative	Moderate	Liberal
(a) Warmth			
Fairness argument	10.59 (23.21, 37)	27.45 (28.10, 74)	42.04 (27.17, 84)
Loyalty argument	9.08 (20.61, 39)	29.45 (29.03, 71)	54.59 (27.24, 87)
(b) Acceptance as president			
Fairness argument	25.73 (37.81, 37)	34.92 (32.26, 74)	60.95 (30.80, 84)
Loyalty argument	10.31 (19.42, 39)	35.25 (33.16, 71)	61.07 (33.77, 87)
(c) Likelihood to vote			
Fairness Argument	10.70 (26.50, 37)	33.88 (38.81, 74)	63.26 (38.32, 84)
Loyalty argument	10.41 (24.87, 39)	36.20 (40.71, 71)	75.98 (31.11, 87)

*Note*. The acceptance as president measure was recoded so that higher values indicate that participants were more willing to accept Clinton as president.

### Warmth

The regression analysis showed a marginally significant interaction effect, Δ*R*^2^ = .01, *F*(2, 386) = 2.43, *p* = .090. Simple-slopes analyses indicated that, as expected, liberal participants perceived Clinton as less warm in the fairness argument condition than in the loyalty argument condition, *b* = −12.55, *t*(386) = −3.06, *p* = .002, 95% CI [−20.61, −4.49]. There was no significant effect of the moral argument condition for either moderates, *b* = −2.00, *t*(386) = −0.45, *p* = .653, 95% CI [−10.76, 6.75], or conservatives, *b* = 1.52, *t*(386) = 0.25, *p* = .805, 95% CI [−10.57, 13.61].

### Acceptance as President

The interaction effect was not significant, Δ*R*^2^ = 0.01, *F*(2, 386) = 1.83, *p* = .162. In addition, simple effects analysis did not provide support for our hypothesis: Liberals in the fairness argument condition did not accept Clinton significantly less as president than liberals in the loyalty argument condition, *b* = −0.12, *t*(386) = −0.02, *p* = .981, 95% CI [−9.74, 9.51]. Additionally, there was no significant effect of the moral argument condition for moderates, *b* = −0.33, *t*(386) = −0.06, *p* = .950, 95% CI [−10.79, 10.12], but there was some evidence that conservatives in the fairness argument condition accepted Clinton more as president than conservatives in the loyalty argument condition, *b* = 15.42, *t*(386) = 2.10, *p* = .036, 95% CI [0.98, 29.87].

### Likelihood to Vote for Clinton

**Figure 2.**
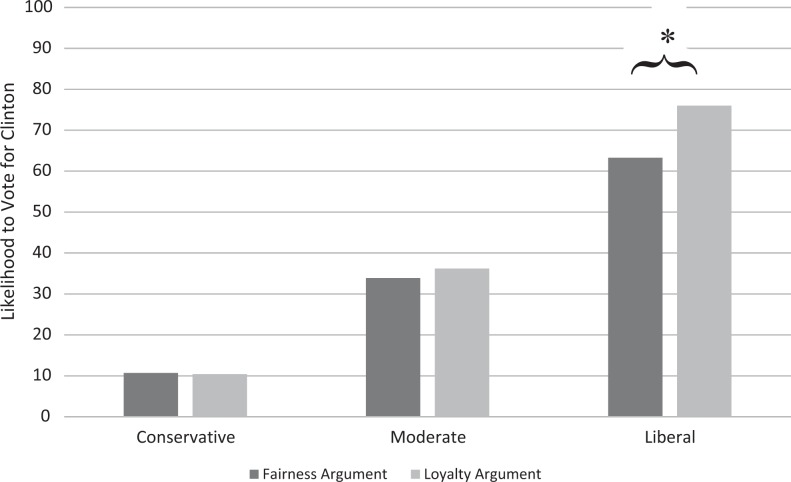
Likelihood to vote for Clinton depending on argument condition and participants' ideology.* **p < .05, **p < .01.

The interaction effect was not significant, Δ*R*^2^ = 0.00, *F*(2, 386) = 1.27, *p* = .282. However, simple-slopes analyses indicated that, as expected, liberal participants were less likely to vote for Clinton in the fairness argument condition than in the loyalty argument condition, *b* = −12.72, *t*(386) = −2.36, *p* = .019, 95% CI [−23.32, −2.11]. There was no significant effect of condition for either moderates, *b* = −2.32, *t*(386) = −0.40, *p* = .692, 95% CI [−13.83, 9.20], or conservatives, *b* = 0.29, *t*(386) = 0.04, *p* = .971, 95% CI [−15.62, 16.20]. These findings are illustrated in Figure 2.

### Moderated Mediation Analyses

Although the interaction effects above were either marginal or not significant, we still chose to conduct moderated mediation analyses in line with Rucker, Preacher, Tormala, and Petty’s (2011) argument that a significant total effect is not a requirement for a significant indirect effect to occur. However, it should be emphasized that such mediation analyses of nonsignificant effects should be interpreted cautiously. For liberals, the effect of experimental condition on the likelihood to vote for Clinton was mediated by warmth, *b* = −10.80, standard error (*SE*) = 3.76, 95% CI [−18.36, −3.84], but not by acceptance as president, *b* = −0.02, *SE* = 1.00, 95% CI [−2.18, 1.84], and the direct effect of experimental condition on likelihood to vote for Clinton was not significant for liberals, *b* = −1.89, *t*(384) = −0.53, *p* = .596, 95% CI [−8.90, 5.12]. For moderates, there was no indirect effect of experimental condition on the likelihood to vote for Clinton, for warmth: *b* = −1.73, *SE* = 4.03, 95% CI [−9.74, 6.20], or for acceptance as president: *b* = −0.06, *SE* = 1.09, 95% CI [−2.14, 2.31], and the direct effect of experimental condition on likelihood to vote for Clinton was not significant for moderates, *b* = −0.53, *t*(384) = −0.14, *p* = .890, 95% CI [−8.02, 6.96]. For conservatives, there was an unexpected indirect effect of experimental condition on the likelihood to vote for Clinton via acceptance as president, *b* = 2.99, *SE* = 1.51, 95% CI [0.72, 6.85], but not for warmth: *b* = 1.31, *SE* = 4.37, 95% CI [−7.21, 10.05]. The direct effect of experimental condition on likelihood to vote for Clinton was not significant for conservatives, *b* = −4.01, *t*(384) = −0.76, *p* = .450, 95% CI [−14.43, 6.41].

## Discussion

The results of Study 2, though in line with our hypotheses, were more complex than the results of Study 1. Although the results of the simple effects analyses were generally consistent with the predictions of a moral reframing account, the predicted interaction effects were nonsignificant for two of our dependent variables and marginally significant for the third dependent variable. Interestingly, we found some evidence for a moral reframing effect for conservatives, which, though not directly hypothesized, is in the direction predicted by a moral reframing account such that conservatives were more persuaded by appeals grounded in the more conservative moral foundation of loyalty.

## General Discussion

Across two studies using the two major candidates from the 2016 U.S. presidential election as targets, we found evidence that moral reframing can be an effective strategy for persuading the electorate about political candidates (for a discussion of the differences between Study 1 and Study 2 see below). As such, the present inquiry extends past research on moral reframing in important ways. Although it has been shown that moral reframing can increase the support of liberals and conservatives for policies that they would usually oppose (e.g., Feinberg & Willer, 2013, 2015), the present research provides the first evidence that moral reframing is also an effective strategy to decrease the attachment of liberals and conservatives to the political candidate of the party they typically support.

Furthermore, the current findings illustrate that despite the fundamental moral differences separating liberals and conservatives (e.g., Graham et al., 2009; Jost, Glaser, Kruglanski, & Sulloway, 2003; Lakoff, 2002), there are ways that people across the ideological spectrum can make their stance understandable to a person from the other side. Although much research has outlined the enormous difficulties involved in fostering productive conversations and collaborations between liberals and conservatives (e.g., Brandt, Reyna, Chamber, Crawford, & Wetherell, 2014; Toner, Leary, Asher, & Jongman-Sereno, 2013), the current research highlights a technique where supporters of political candidates are responsive to criticism about their favored candidate, and as a result, decreases the distance between liberals and conservatives.

The effectiveness of moral reframing raises the question of whether campaigns, pundits, and everyday people actually employ this technique to affect people’s opinion about political candidates. In a first attempt to investigate this question, we asked liberal Clinton supporters and conservative Trump supporters to write arguments aimed at convincing those who endorse the other candidate as to why they should instead oppose him or her. In addition, we investigated the content of YouTube videos opposing Hillary Clinton or Donald Trump. Our results suggested that conservatives used morally reframed arguments more than liberals (cf. Haidt, 2012). However, this evidence should be viewed as only preliminary and fodder for future research (for more information, see Supplemental Material).

Overall, the present research had several important limitations. First, the support for a moral reframing effect was generally stronger in Study 1 than in Study 2. This difference could be driven by a number of factors. For instance, the content of the fairness and loyalty messages in the two studies was different, opening up the possibility that the quality or intensity of the arguments may have differed across studies. Another possibility could be that Trump’s candidacy might be more strongly associated with issues of morality than Clinton’s candidacy. Therefore, it might be easier to stimulate people’s moral intuitions relating to Trump. Future research could potentially address this question by examining archival data (e.g., open-ended responses collected in polls), testing the extent to which beliefs about Trump and Clinton reflect individuals’ core moral values and convictions (cf. Skitka & Bauman, 2008).

Furthermore, although we found support for the effectiveness of moral reframing with regard to both attitudes toward the candidates and behavioral intentions, we did not use measures of real behavior (cf. Wolsko et al., 2016). Potentially tracking participants’ actual voting behavior after exposure to reframed messages would be a promising route for future research. Furthermore, we only examined messages that appealed to the fairness and loyalty foundation. We chose these foundations as our examination of popular media suggested much of the information published about the candidates fit within a fairness or loyalty argument frame. Even so, it is an open question for future research how influential arguments couched in the other moral foundations might be.

Additionally, our two studies found an only partially consistent pattern in their moderated mediation analyses. In addition, the results of the second study should be considered with caution considering the nonsignificant interaction effects. Future research is needed to explore the mechanisms underlying the moral reframing effect in the political elections domain. Finally, in the present research, we did not have control conditions, and therefore it is impossible to know for sure which of the two conditions in the studies caused the persuasion effects we found. However, past research has used control conditions and found that the effect is in line with the moral reframing hypothesis (Feinberg & Willer, 2013, 2015), and as such, we feel confident that the effects we found were due to the morally reframed conditions.

Overall, our findings add to the growing body of research demonstrating how important it is to recognize and understand the moral values of those who take an opposing political position (Feinberg & Willer, 2013, 2015; Kidwell et al., 2013; Wolsko et al., 2016). As a whole, this literature highlights that the more individuals take the moral perspective of those who do not agree with them into consideration, the more successful they will be at reaching those individuals. The present research demonstrates that this is even the case in the context of one of the most politically polarizing events—political campaigns.

## Supplemental Material

SPPS729408_suppl_mat - Morally Reframed Arguments Can Affect Support for Political Candidates Click here for additional data file. SPPS729408_suppl_mat for Morally Reframed Arguments Can Affect Support for Political Candidates by Jan G. Voelkel, and Matthew Feinberg in Social Psychological and Personality Science
