# Supplementary material for: Morally Reframed Arguments Can Affect Support for Political Candidates
Source: Soc Psychol Personal Sci. 2017 Sep 28;9(8):917–24. doi: 10.1177/1948550617729408 (PMC6295651; doi:10.1177/1948550617729408)
Supplement: SPPS729408_suppl_mat - Morally Reframed Arguments Can Affect Support for Political Candidates [file SPPS729408_suppl_mat.docx]

**Supplementary Online Materials**

Arguments used in Studies 1-2

*Study 1*

*Fairness argument*

Donald Trump Discriminates Against Muslims

Donald Trump openly discriminates against Muslims threatening their rights to be treated with fairness and equality. He has suggested to temporarily ban Muslims from certain countries from entering the United States and his unfair statements are a breeding ground for prejudice towards Muslims. His unfair actions exclude Muslims from their chance to become part of American society.

No matter what your political position, Donald Trump's discrimination of foreigners based on their religious beliefs is unacceptable.

*Loyalty argument*

Trump First, America Second

Donald Trump has repeatedly behaved disloyally towards our country to serve his own interests. For instance, during the Vietnam War, he dodged the draft to follow his father into the development business. Recently, he encouraged Russian agents to commit espionage against the US to find information that could help him to win the presidential election.
No matter what your political position, Donald Trump's disloyalty towards the United States to serve his own interests is unacceptable.

*Study 2*

*Fairness argument*

**Hillary *First*, Fairness *Second***

Hillary Clinton has taken advantage of unfair support throughout her political career. While so many Americans have suffered during the recent recession that the Wall Street Banks helped cause, Clinton has accepted millions of dollars from them in exchange for giving a few speeches. And, even though Clinton claims to be a strong advocate for gender equality, her actions do not always demonstrate this. For example, in her own foundation, male executives earn 38% more than female executives! Also, the Clinton Foundation has received millions of dollars from Saudi Arabia, even though it is well-known that women in Saudia Arabia^[[1]](#footnote-1)^ are treated like property -- they cannot choose the clothes they wear, play sports, or even drive a car without risking persecution.
No matter what your political position, it is clear that Hillary Clinton is willing to sacrifice fairness and equality to achieve her own goals.

*Loyalty argument*

**Hillary *First*, America *Second***

Hillary Clinton has failed to perform her civic duty and, as a result, has put America's standing in the world in jeopardy. During her time as the Secretary of State, she failed our ambassador and soldiers in Benghazi, resulting in terrorists taking over America's consulate.  She used her private email server for official communications making them highly vulnerable to hackers from places like Russia and China, who may have stolen American secrets right from under our noses. Furthermore, although 19 of the 21 Sept 11th hijackers were from Saudia Arabia^6^, Clinton supported the sale of 80 fighter jets to Saudi Arabia, which have since been used in a war that allowed al Qaeda to seize more territory. 
No matter what your political position, it is clear that Hillary Clinton is willing to risk the standing of our nation to achieve her own goals.

Robustness checks in Studies 1-2

*Study 1*

*Exclusion procedures.* The results were robust for different exclusion procedures. Not excluding participants due to the attention check (final n = 401) did not change the results substantively. Excluding participants whose summaries were judged as inadequate by at least one rater (final n = 393) also did not change the results substantively.

*Heteroscedasticity.* An inspection of a plot of studentized residuals versus unstandardized predicted values indicated that the assumption of homoscedasticity might be violated. So, we also ran the analyses with heteroscedasticity-consistent standard errors. The results did not change substantively except for the following differences. For warmth, the interaction effect and the effect for conservatives became both marginally significant. For likelihood to vote for Trump, the effect for liberals became significant.

*Ideology as continuous variable.* Treating ideology as a continuous moderator also leads to significant interaction effects in the predicted direction for the three dependent variables (warmth, acceptance as president, likelihood to vote; *p*s < .05).

*Study 2*

*Exclusion procedures.* As in Study 1, the results were robust for different exclusion procedures. Not excluding participants due to the attention check (final *n* = 405) did not change the results substantively except for the following differences. For warmth, the interaction effect became non-significant. For acceptance as president, the effect of condition for conservatives became marginally significant. Excluding participants whose summaries were judged as inadequate by at least one rater (final *n* = 389) did not change the results substantively except for the following difference. For warmth, the interaction effect became significant.

*Heteroscedasticity.* As an inspection of a plot of studentized residuals versus unstandardized predicted values indicated that the assumption of homoscedasticity might be violated, we reran the analyses with heteroscedasticity-consistent standard errors. The results did not change substantively.

*Ideology as continuous variable.* Treating ideology as a continuous moderator leads to significant interaction effects in the predicted direction for warmth (*p* < .05), and two interaction effects close to significance for acceptance as president and likelihood to vote (*p*s < .14).

Investigation of Usage of Moral Reframing

*Laypeople’s arguments opposing Donald Trump*

*Participants.* One hundred and seventy-six liberals recruited from the Amazon Mechanical Turk website who indicated that they opposed Donald Trump more than Hillary Clinton completed the study.

*Procedure.* Participants learned that this study was about creating a message about a presidential candidate. Next, they filled out two filter questions. First, they indicated which of the two presidential candidates (Donald Trump and Hillary Clinton) they opposed more. Second, they filled out the same measure of political ideology as in Studies 1 and 2. Only liberals who opposed Donald Trump more strongly were filtered into this study.

Next, participants were asked to write a persuasive argument “aimed at convincing conservative Americans who support Donald Trump for president why they should oppose Donald Trump”. Participants were further instructed not to write about the advantages of other candidates but rather to focus on a critique of Donald Trump. They were also motivated by a bonus of $50 for the best persuasive argument. Afterwards, participants completed the same measures of attitudes towards Donald Trump and Hillary Clinton as in Studies 1 and 2 (warmth, acceptance as president, and likelihood to vote) and a small demographic questionnaire.

*Coding.* Five research assistants (blind to the hypotheses) coded each of the arguments on (a) to what extent the argument appealed to the moral values related to each of the five moral foundation and (b) to what extent the argument attacked the moral values related to each of the five moral foundations. All codings were on a scale from 1 (not at all) to 5 (a whole lot)^[[2]](#footnote-2)^.

The reliability of the ratings of the research assistants was low to medium for the appealing ratings (.38 < αs < .61), except for the authority/subversion dimension (α = .11). The reliability of the ratings of the research assistants was very low for the attacking ratings (-.03 < αs < .17). Therefore, we focused on the appealing ratings in the remaining discussion of this study. We averaged the appealing scores of the five raters for each of the five moral foundation so that we had five dependent variables: the extent to which arguments criticizing Donald Trump appealed to a) care/harm values, b) fairness/cheating values, c) loyalty/betrayal values, d) authority/subversion values, e) sanctity/degradation values.

*Analysis strategy.* We conducted a repeated measures ANOVA with moral foundation (care/harm values vs. fairness/cheating values vs. loyalty/betrayal values vs. authority/subversion values vs. sanctity/degradation values) as within-subjects factor. We used the Greenhouse-Geister correction for violations of sphericity.

*Results.* Our hypothesis that liberals would use the two liberal foundations (individualizing foundations) rather than the three conservative foundations (binding foundations) to convince conservatives to oppose Donald Trump was supported. The repeated measures ANOVA showed a significant main effect for moral foundation, *F*(3.40, 594.84) = 184.52, *p* < .001, partial *η*² = .51. Liberal participants used the individualizing foundations more often than the binding foundations. Specifically, the care/harm foundations was used significantly more than the fairness/cheating foundation, *F*(1, 175) = 20.53, *p* < .001, partial *η*² = .10. In turn, the fairness/cheating foundation was used significantly more than the loyalty/betrayal foundation, *F*(1, 175) = 8.04, *p* = .005, partial *η*² = .04. The loyalty/betrayal foundation was used significantly more than the authority/subversion foundation, *F*(1, 175) = 27.70, *p* < .001, partial *η*² = .14. The authority/subversion foundation was used significantly more than the sanctity/degradation foundation, *F*(1, 175) = 241.73, *p* < .001, partial *η*² = .58. In short, liberal participants used the two individualizing foundations significantly more often to convince conservatives to oppose Donald Trump than the three binding foundations.

*Laypeople’s arguments opposing Hillary Clinton*

*Participants.* One hundred and six conservatives recruited from the Amazon Mechanical Turk website^[[3]](#footnote-3)^ who indicated that they opposed Hillary Clinton more than Donald Trump completed the study. As two arguments were consistently judged as arguments that do not oppose Hillary Clinton by five research assistants, these two cases were excluded and the final sample size consisted of 104 arguments.

*Procedure.* The procedure was the same for the arguments opposing Donald Trump except that this time only conservatives who opposed Hillary Clinton more strongly were filtered into the study. Furthermore, the argument participants were asked to create was aimed at convincing liberal participants of why they should oppose Hillary Clinton.

*Codings.* The coding procedure was identical to the coding procedure for the arguments opposing Donald Trump.

Again, the reliability of the ratings of the research assistants was low to medium for the appealing ratings (.35 < αs < .63). The reliability of the ratings of the research assistants was very low for the attacking ratings (-.03 < αs < .13). Therefore, we focused on the appealing ratings in the remaining discussion of this study.

*Analysis strategy.* We used the same analysis strategy as above.

*Results.* Our hypothesis that conservatives would use the binding foundations rather than the individualizing foundations to convince liberals to oppose Hillary Clinton was not supported. The repeated measures ANOVA showed a significant main effect for moral foundation, *F*(3.53, 364.09) = 139.96, *p* < .001, partial *η*² = .58. Conservative participants did not use the binding foundations more often than the individualizing foundations. Specifically, the fairness/cheating foundations was used significantly more than the loyalty/betrayal foundation, *F*(1, 103) = 28.45, *p* < .001, partial *η*² = .22. The loyalty/betrayal foundation was used to a similar amount as the care/harm foundation, *F*(1, 103) = 2.21, *p* = .140, partial *η*² = .02. The care/harm foundation was used significantly more than the authority/subversion foundation, *F*(1, 103) = 54.80, *p* < .001, partial *η*² = .35. The authority/subversion foundation was used significantly more than the sanctity/degradation foundation, *F*(1, 103) = 94.22, *p* < .001, partial *η*² = .48. In short, conservative participants mostly used the two individualizing foundations and the loyalty/betrayal foundation to convince liberals to oppose Hillary Clinton.

*YouTube videos opposing Donald Trump*

*Sample.* We searched for videos on youtube.com with the following terms "Hillary Clinton Donald Trump ad", "Donald Trump Hillary Clinton ad", "Donald Trump ad", and "Hillary Clinton ad." We only included videos that were no longer than two minutes and had at least 10 000 views (on September 7, 2016). Then we asked two research assistants (blind to the hypotheses) to code all videos that matched these criteria on whether these videos were professionally made, whether the ad was a critique or praise for one of the two candidates, who was the target of the ad, and who was the source of the ad. Videos that were professionally made, were critique ads, and had only Donald Trump as target constituted the sample size for Study 3 (*n* = 62). However, seven of these videos had to be removed as they were deleted from youtube.com before the coding procedure started. So, the final sample size for this study consisted of 55 videos.

*Codings.* The coding procedure was identical to the procedure described above.

Again, the reliability of the ratings of the research assistants was low to medium for the appealing ratings (.33 < αs < .71). The reliability of the ratings of the research assistants was very low for the attacking ratings (-.07 < αs < .17). Therefore, we focused on the appealing ratings in the remaining discussion of this study.

*Analysis strategy.* We used the same analysis strategy as above.

*Results.* Our hypothesis that the campaigns in the video would use the individualizing foundations rather than the binding foundations to criticize Donald Trump was supported. The repeated measures ANOVA showed a significant main effect for moral foundation, *F*(3.52, 190.33) = 23.00, *p* < .001, partial *η*² = .30. There was no significant difference between the most-often used care/harm foundation and the second most-often used fairness/cheating foundation, *F*(1, 54) = 0.25, *p* = .622, partial *η*² = .00, as well as between the fairness/cheating foundation and the third most often used loyalty/betrayal foundation, *F*(1, 54) = 0.35, *p* = .554, partial *η*² = .01. However, the loyalty/betrayal foundation was used significantly more than the authority/subversion foundation, *F*(1, 54) = 6.82, *p* = .012, partial *η*² = .11. The authority/subversion foundation was used significantly more than the sanctity/degradation foundation, *F*(1, 54) = 32.93, *p* < .001, partial *η*² = .38. In short, YouTube videos that opposed Donald Trump were mostly based on the two individualizing foundations, to a slightly lower extent on the loyalty/betrayal foundation, and to a much lower extent on the two other binding foundations.

*YouTube Videos opposing Hillary Clinton*

*Sample.* We used the same search strategy as above, but included only videos that had only Hillary Clinton as target (*n* = 54). However, one of these videos had to be removed from youtube.com as it was deleted before at least three coders could code it. So, the final sample size for this study consisted of 53 videos.

*Codings.* The coding procedure was identical to the procedure used above.

Again, the reliability of the ratings of the research assistants was low to medium for the appealing ratings (.33 < αs < .76). The reliability of the ratings of the research assistants was partly very low for the attacking ratings (-.03 < αs < .61). Therefore, we focused on the appealing ratings in the remaining discussion of this study.

*Analysis strategy.* We used the same analysis strategy as above.

*Results.* Our hypothesis that the campaigns in the video would use the binding foundations rather than the individualizing foundations to criticize Hillary Clinton was partly supported. The repeated measures ANOVA showed a significant main effect for moral foundation, *F*(2.53, 131.36) = 40.03, *p* < .001, partial *η*² = .43. The loyalty/betrayal foundation was used most often, but the difference to the second most often used fairness/cheating foundation was not significant, *F*(1, 52) = 0.62, *p* = .436, partial *η*² = .01. However, the difference between the loyalty/betrayal foundation and the third most often used care/harm foundation was significant, *F*(1, 52) = 4.32, *p* = .043, partial *η*² = .08. The difference between the fairness/cheating foundation and the care/harm foundation was not significant, *F*(1, 52) = 0.49, *p* = .489, partial *η*² = .01. Further, the care/harm foundation was used significantly more than the authority/subversion foundation, *F*(1, 52) = 41.17, *p* < .001, partial *η*² = .44. The authority/subversion foundation was used significantly more than the sanctity/degradation foundation, *F*(1, 52) = 24.15, *p* < .001, partial *η*² = .32. In short, YouTube videos that opposed Hillary Clinton were mostly based on the loyalty/betrayal foundation and the two individualizing foundations, but to a much lower extent on the two other binding foundations.

1. The argument included a small spelling mistake. Instead of “Saudia Arabia” it should have been “Saudi Arabia”. [↑](#footnote-ref-1)
2. One coder accidentally coded on a scale from 0 to 5. We recoded these ratings using the following formula (Old_Coding/5*4 +1) so that the codings were also between 1 and 5. [↑](#footnote-ref-2)
3. Note that in order to increase the number of conservatives, we used the following procedure: From a prescreened sample of about 50 000 participants, we selected those who scored either 6 or 7 (out of 7) on political ideology (7 being extremely conservative). Then, we randomly selected approximately 1000 of these participants and directly emailed them in order to invite them to participate in the study. After approximately one day of them participating, we then opened the study up to the full MTurk population. [↑](#footnote-ref-3)
